# Analysis of porcine *IGF2* gene expression in adipose tissue and its effect on fatty acid composition

**DOI:** 10.1371/journal.pone.0220708

**Published:** 2019-08-08

**Authors:** Lourdes Criado-Mesas, Maria Ballester, Daniel Crespo-Piazuelo, Anna Castelló, Rita Benítez, Ana Isabel Fernández, Josep M. Folch

**Affiliations:** 1 Departament de Genòmica Animal, Centre de Recerca en Agrigenòmica (CRAG), CSIC-IRTA-UAB-UB, Barcelona, Spain; 2 Departament de Genètica i Millora Animal, Institut de Recerca y Tecnologia Agraroalimentàries (IRTA), Caldes de Montbui, Spain; 3 Departament de Ciència Animal i dels Aliments, Facultat de Veterinària, UAB, Bellaterra, Spain; 4 Departamento de Mejora Genética Animal, Instituto Nacional de Investigación y Tecnología Agraria y Alimentaria (INIA), Madrid, Spain; Universita degli Studi di Bologna, ITALY

## Abstract

*IGF2*:*g*.*3072G>A* polymorphism has been described as the causal mutation of a maternally imprinted QTL for muscle growth and fat deposition in pigs. The objective of the current work was to study the association between the *IGF2*:*g*.*3072G>A* polymorphism and the *IGF2* gene expression and its effect on fatty acid composition in adipose tissue in different pig genetic backgrounds. A *cis*-eQTL region associated with the *IGF2* mRNA expression in adipose tissue was identified in an eGWAS with 355 animals. The *IGF2* gene was located in this genomic interval and *IGF2g*.*3072G>A* was the most significant SNP, explaining a 25% of the gene expression variance. Significant associations between *IGF2*:*g*.*3072G>A* polymorphism and oleic (C18:1(n-9); p-value = 4.18x10^-07^), hexadecanoic (C16:1(n-9); p-value = 4.04x10^-07^), linoleic (C18:2(n-6); p-value = 6.44x10^-09^), α-linoleic (C18:3(n-3); p-value = 3.30x10^-06^), arachidonic (C20:4(n-6); p-value = 9.82x10^-08^) FAs and the MUFA/PUFA ratio (p-value = 2.51x10^-9^) measured in backfat were identified. Animals carrying the *A* allele showed an increase in *IGF2* gene expression and higher PUFA and lower MUFA content. However, in additional studies was observed that there could be other proximal genetic variants affecting FA composition in adipose tissue. Finally, no differences in the *IGF2* gene expression in adipose tissue were found between heterozygous animals classified according to the *IGF2*:*g*.*3072G>A* allele inherited from the father (*A*^*P*^*G*^*M*^ or *A*^*M*^*G*^*P*^). However, pyrosequencing analysis revealed that there is imprinting of the *IGF2* gene in muscle and adipose tissues, with stronger differences among the paternally and maternally inherited alleles in muscle. Our results suggested that *IGF2*:*g*.*3072G>A* polymorphism plays an important role in the regulation of *IGF2* gene expression and can be involved in the fatty acid composition in adipose tissue. In both cases, further studies are still needed to deepen the mechanism of regulation of *IGF2* gene expression in adipose tissue and the *IGF2* role in FA composition.

## Introduction

Over the last few years there has been a highlighted interest in identifying genes that improve meat quality. The nutritional value of meat and its quality is determined by several factors, including the intra-muscular fat (IMF) content and its fatty acid (FA) composition. Fat tissue firmness, shelf life, flavour, tenderness and juiciness [1] are influenced by FA composition, which is also involved in both meat nutritional traits and common diseases such as obesity and diabetes [2].

A paternally expressed Quantitative Trait Locus (QTL) for muscle growth and backfat (BF) thickness was identified in pig chromosome 2 (SSC2), in a genomic region containing the *insulin-like growth factor 2* (*IGF2*) gene [3,4]. *IGF2* is a maternally imprinted gene which promotes growth and plays an important role in proliferation, differentiation and apoptosis of cells in different tissues [5,6]. Moreover, IGF2 dysfunctions are involved in metabolic disorders, such as diabetes and obesity among others [7]. Latterly, IGF2 has been proposed as a physiological regulator of preadipocyte growth, metabolism and body fat composition in humans [8,9], although regulation of the *IGF2* gene is still uncertain.

Some years later, the polymorphism *g*.*3072G>A* located in the intron 3 of the *IGF2* gene was described as the causal mutation for this QTL, which increases muscle growth and heart size and reduces subcutaneous fat deposition [10]. The mutation is located in a well-conserved CpG island that is hypomethylated in skeletal muscle and abrogates the binding site for ZBED6, a nuclear factor which repress *IGF2* transcription, leading to a 3-fold up-regulation of *IGF2* expression in skeletal muscle [11]. The *IGF2*:*g*.*3072G>A* polymorphism has been associated with *IGF2* expression in muscle, but not in liver [10] and adipose [12] tissues, indicating a tissue-dependent regulation of *IGF2* gene expression.

The causal mutation for this QTL is widespread in different breeds [13] and it contributes to the improvement of porcine production, explaining 15–30% of the phenotypic variation in muscle mass and 10–20% of the variation in BF thickness [3,4]. The effects of this mutation on several growth traits have also been identified in different populations. For example, in a Large White commercial population and in an Iberian x Landrace F2 cross the *IGF2* polymorphism was associated with BF thickness, carcass weight, *longissimus* muscle area, ham weight and shoulder weight traits [14].

Furthermore, an association between the *IGF2* gene expression and the percentage of IMF, in which animals with a high gene expression presented greater IMF content in skeletal muscle, has been described [15]. Another study showed that the mutation has an effect on both carcass and ham conformation and they detected an increase in monounsaturated FA (MUFA) and a decrease in polyunsaturated FA (PUFA) content in hams of pigs carrying the *A* allele [16]. However, there is a lack of literature analysing the effect of the *IGF2*:*g*.*3072G>A* polymorphism on FA composition measured in adipose tissue.

The aim of this work was to study the association between the *IGF2*:*g*.*3072G>A* polymorphism and the *IGF2* gene expression in adipose tissue to better understand 1) the *IGF2* gene expression regulation in adipose tissue and 2) the effect of the *IGF2* gene on adipose tissue FA composition.

## Material and methods

### Animal material

A total of 355 animals belonging to different experimental backcrosses, BC1_LD (25% Iberian and 75% Landrace), BC1_DU (25% Iberian and 75% Duroc) and BC1_PI (25% Iberian and 75% Pietrain), were analyzed. This set of animals from three different backcrosses was named 3BCs. All animals were maintained under intensive conditions and feeding was *ad libitum* with a cereal-based commercial diet. Animal procedures were performed according to the Spanish Policy for Animal Protection RD1201/05, which meets the European Union Directive 86/609 about the protection of animals used in experimentation. The experimental protocol was approved by the Ethical Committee of IRTA (Institut de Recerca i Tecnologia Agroalimentàries).

BF samples were taken between the third and the fourth ribs, collected at slaughter in liquid nitrogen and stored at -80°C until analysis. Genomic DNA was extracted from diaphragm tissue samples using the phenol-chloroform method [17].

### Phenotypic data

Composition of 17 FAs in the C:12 and C:22 range in BF adipose tissue was determined by gas chromatography of methyl esters [18]. Afterwards, the percentage of the content of each FA was calculated in addition to the overall percentage of saturated FAs (SFA), MUFA and PUFA. In addition, ratios of FA as indices for desaturation and elongation were determined. BF thickness was measured between the 4^th^ and the 5^th^ ribs.

### Genotyping

Animals from BC1_LD and BC1_PI were genotyped with Porcine SNP60K BeadChip (Illumina, San Diego, USA) and BC1_DU animals with Axiom Porcine Genotyping Array (Affymetrix, Inc.).

Common Single Nucleotide Polymorphisms (SNPs) in both arrays were mapped against the *Sus scrofa 11*.*1* assembly and Plink software [19] was used afterwards to remove markers that showed a minor allele frequency (MAF) less than 5% and SNPs with more than 5% of missing genotypes. After filtering, a total of 38,424 SNPs were retained for association studies. In addition, the *IGF2*:*g*.*3072G>A* polymorphism was genotyped using a pyrosequencing protocol previously described [10] and a SNP located in the predicted 3’ UTR region of the gene (*ENSSSCT00000039341*.*1*:*c*.*1469990C>T*) was genotyped using Taqman OpenArrayTM genotyping plates custom-designed in a QuantStudioTM 12K flex Real-Time PCR System (ThermoFisher Scientific).

### Gene expression

Reverse transcription quantitative real time-PCR (RT-qPCR) was used to study *IGF2* gene expression in a total of 355 animals from BC1_LD (n = 114), BC1_DU (n = 122) and BC1_PI (n = 119) in BF adipose tissue. In addition, the *Longissimus dorsi* (LD) muscle *IGF2* expression was analysed in 14 animals corresponding to BC1_LD (n = 7) and BC1_DU (n = 7). Total RNA was obtained using the RiboPure kit (Ambion), following the producer’s recommendations. RNA was quantified using the NanoDrop ND-1000 spectrophotometer (NanoDrop products) and the RNA integrity was assessed by Agilent Bioanalyzer-2100 (Agilent Technologies). One microgram of total RNA was reverse-transcribed into cDNA using the High-Capacity cDNA Reverse Transcription kit (Applied Biosystems) using random hexamer primers in 20 μl reactions, following the manufacturer’s instructions. Minus reverse transcription polymerase controls were also included to test for residual genomic DNA amplification. Primers for *IGF2* and two reference genes, *actin beta* (*ACTB*) and *TATA box binding protein* (*TBP*) (S1 Table), were designed using PrimerExpress 2.0 software (Applied Biosystems) [20].

Gene expression quantification in BF adipose tissue samples was performed in a QuantStudio 12K Flex Real-Time PCRSystem (ThermoFisher Scientific) using a 384-well plate and each sample was analyzed per triplicate. PCR amplifications were done in a final volume of 15 μl, including: 7,5 μl of SYBR Select Master Mix (ThermoFisher Scientific), 300 nM of each primer and 3,75 μL of a 1:25 cDNA dilution. The PCR thermal cycle was: 2 min at 50°C, 10 min at 95°C, 40 cycles of 15 sec at 95°C and 1 min at 60°C. Moreover, a melting profile (95°C for 15 sec, 60°C for 15 sec and a gradual increase in temperature with a ramp rate of 1% up to 95°C) was added following the thermal cycling protocol, to assess for the specificity of the reactions. RT-qPCR efficiency for each assay was controlled using relative standard curves generated from a pool of cDNA from all samples serially diluted 5 fold. Data was collected and analyzed using the ThermoFisher Cloud software 1.0 (Applied Biosystems) applying the 2^-ΔΔCt^ [21] method for relative quantification (RQ) and using the lowest expression sample as calibrator.

Gene expression quantification in LD samples was performed in a 48.48 Microfluidic Dynamic Array IFC Chip (Fluidigm) in a BioMark System following a previously described protocol [22]. Data was collected and analysed using Fluidigm Real-Time PCR analysis software 3.0.2 (Fluidigm) and DAG Expression software 1.0.4.11 [23] respectively, applying the relative standard method curve.

Normalization of data was checked through Shapiro-Wilk test in R (https://r-project.org/) and log2 transformation was applied. A linear model (lm) was used also in R for test sex and breed effects [24].

### Differential allelic expression quantification by pyrosequencing

A subset of 14 animals were selected based on their deduced paternally-inherited alleles and complete linkage disequilibrium between the *IGF2*:*g*.*3072G>A* and the *IGF2* 3’ UTR (*ENSSSCT00000039341*.*1*:*c*.*1469990C>T*) polymorphisms, being all heterozygous for both variants. Seven animals carried the paternally derived haplotype *IGF2*:*g*.*3072 A*—*ENSSSCT00000039341*.*1*:*c*.*1469990 C* and 7 animals the alternative paternally derived *IGF2*:*g*.*3072 G—ENSSSCT00000039341*.*1*:*c*.*1469990 T* haplotype. Hence, analysis of the allelic expression at *ENSSSCT00000039341*.*1*:*c*.*1469990C>T* variant allowed us to infer the relative expression of *IGF2*:*g*.*3072G>A* alleles. Pyrosequencing analyses were performed in both muscle and adipose tissues.

A 114-bp fragment of the 3’-UTR region of *IGF2* gene containing the *ENSSSCT00000039341*.*1*:*c*.*1469990C>T* polymorphism was amplyfied using the following primers: Forward primer 5’-CACGCTCGCAGCTCTCTT-3’, Reverse primer 5’-[biotin]CCCCCAGAAAGCTCGGAG-3’ and pyrosequenced with primer 5’-CTCGCAGCTCTCTTG-3’.

RNA samples were treated with the Turbo DNA-free kit (Invitrogen, ThermoFisher Scientific) following manufacturer’s instructions before reverse transcription. Amplification of cDNA samples was done using the GC RICH PCR system (Roche). Reactions included 1.5 mM of MgCl2, 200 μM of dNTP, 0.3 μM of each primer, 1U of GC-rich enzyme mix, 0.5 M GC-rich resolution solution and 2 μl of cDNA diluted 1:2 in a final volume of 25 μl. The thermal profile was 95°C for 3 min, 40 cycles at 95°C for 30 sec, 58°C for 30 sec and 72°C for 45 sec for the first 10 cycles and 5 more sec for each cycle in addition and a final extension step of 7 min at 72°C. We tested whether amplification of genomic DNA was circumvented by RNA treatment with DNase using RNA not reverse-transcribed as a template.

The biotinylated PCR products were checked in high resolution agarose gels and analysed by pyrosequencing at the Sequencing and Functional Genomics Service of the Instituto Aragonés de Ciencias de la Salud (IACSs) with a PSQ 96MA system equipment (Biotage). Pyrosequencing data were analysed and quantified using the AQ mode of PSQ 96MA 2.1. software (Pyrosequencing QIAGEN). Calibration samples were prepared by mixing homozygous genomic DNA samples (TT or CC) at different proportions to check the precision of the assay in estimating allele-specific frequencies.

### Genome-wide association analysis for adipose tissue *IGF2* gene expression

Genomic association studies between gene expression values of *IGF2* and SNPs genotypes (eGWAS) were performed through a linear mixed model using GEMMA software [25]:

$$y=W\alpha +x\beta +u+\varepsilon ;\;u∼MVN_{n}(0,\lambda \tau^{-1}K),\varepsilon ∼MVN_{n}(0,\tau^{-1}I_{n}),$$

in which: y was the vector of phenotypes for n individuals; W is a matrix n×c of covariates (fixed effects) that includes a column of ones, sex (2 levels), backcross (3 levels), and batch (9 levels); α is a c vector with corresponding coefficients, including the intercept; x is an n vector with the marker genotypes; β is the size of the marker effect, u is an n vector of random effects (additive genetic effects), ε is an n vector of errors. The random effects vector is assumed to follow a normal multivariate n-dimensional distribution (MVN_n_) where τ^-1^ is the variance of residual errors; λ is the quotient between the two components of variance; K is an n×n matrix of kinship calculated from the autosomal SNPs. The vector of errors is assumed to follow a distribution MVN_n_, where I_n_ is an n×n identity matrix.

GEMMA software calculates the Wald statistical test and the P-value for each SNP comparing the null hypothesis that the SNP has no effect versus the alternative hypothesis that the SNP effect is different from zero. The FDR (False Discovery Rate) method of Benjamini and Hochberg [26] was used for the correction of multiple tests with the *p*.*adjust* function of R.

### Gene annotation

The significantly associated SNPs were mapped in the *S*. *scrofa 11*.*1* assembly and were annotated with the Ensembl Genes 91 Database using VEP software [27]. The genomic eQTL intervals considering ±1 Mb around the candidate chromosomal regions were annotated using BioMart software [28].

The SNPs identified were classified as *cis* when they were located within 1 Mb from the gene analysed and as *trans* when they were located elsewhere in the genome. Significant SNPs located less than 10 Mb apart were considered as belonging to the same genomic interval.

### Association analysis for adipose tissue fatty acid composition

The linear mixed model previously described for eGWAS, adding carcass weight as a covariate, was carried out to study the association among 2,431 SSC2 SNPs genotypes and FA composition measured in BF tissue in 341 animals using GEMMA software [25].

Correlation analyses were done to better understand the relationship between gene expression and phenotypes. Gene expression was corrected by sex (two levels), backcross (three levels), and batch (nine levels) effects, and the FA composition was adjusted for sex, backcross, batch, and carcass weight. The corrected values of FA composition and gene expression were used to obtain the Pearson pairwise correlations.

### Imprinting analysis

Paternal allele of 355 animals was deduced from progenitor’s genotypes. An imprinting model of *IGF2* expression in BF was tested using a linear model (*lm*) in R, adjusting for sex, backcross, and batch as fixed effects. A comparison between this model and additive model was performed. The same models were tested with FA composition.

## Results and discussion

### Genome wide association study of adipose tissue *IGF2* gene expression

**Fig 1:**
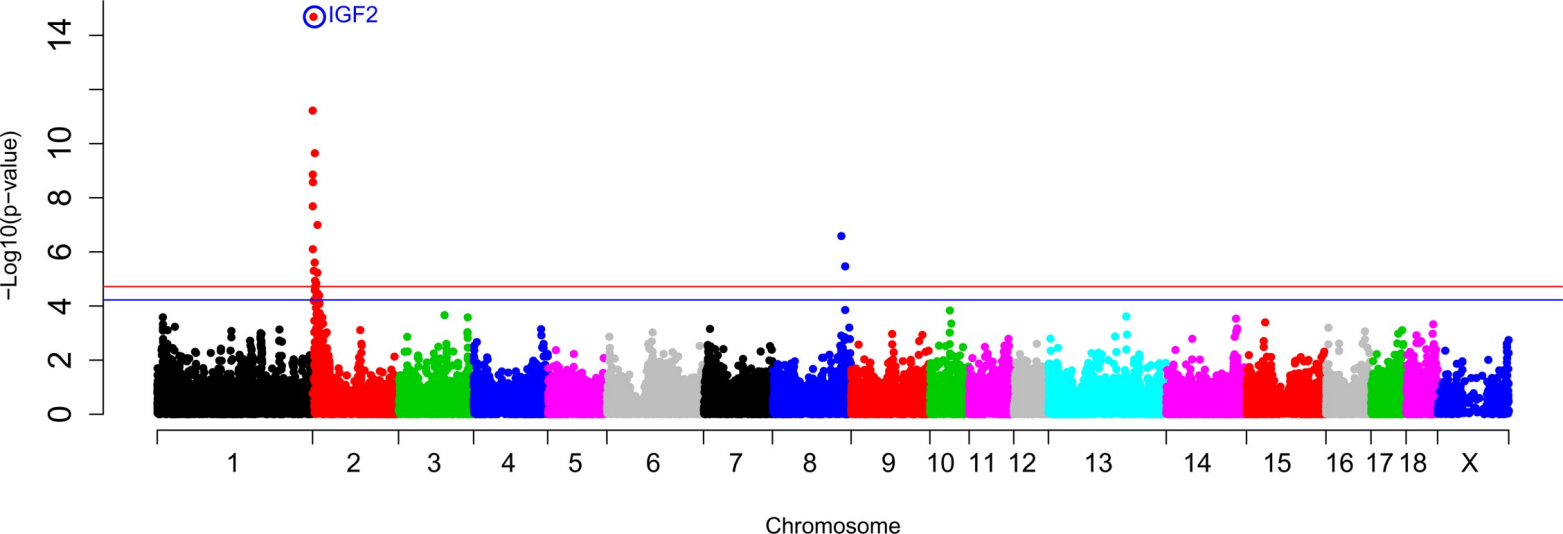
GWAS plot of *IGF2* gene expression in adipose tissue in the 3BCs animals. Chromosome positions in Mb based on *S*. *scrofa 11*.*1* assembly of the pig genome are represented in the X-axis and the–log10 (p-value) is on the Y-axis. The red horizontal line indicates the genome-wide significant level (FDR-based *q*-value < 0.05) and the blue horizontal line represents the genome-wide suggestive level (FDR-based *q*-value < 0.1). The *IGF2*:*g*.*3072G>A* polymorphism is circled and labelled as IGF2 in colour blue.

An eGWAS was performed among the genotypes of 38,425 SNPs, including *IGF2*:*g*.*3072G>A*, and the *IGF2* mRNA expression values in BF adipose tissue of 355 animals from all three backcrosses (3BCs) (Fig 1).

Two chromosomal regions (eQTLs) on SSC2 and SSC8 presented significant associations with the *IGF2* gene expression in adipose tissue using an additive model (Table 1).

**Table 1:** Significant eQTLs for adipose tissue *IGF2* gene expression in the 3BCs animals.

Region	Chr	Start-End Positions	Size (Mb)	SNPs N	Start-End SNPs	Most Significant SNP	P-value	Type of eQTL	Candidate genes*
1	2	0,145,257–11,580,559	11.43	21	*rs81306755- rs81278022*	*IGF2*	2.07E-15	*cis/trans*	*IGF2* and *SF1*
2	8	121,609,989–128,426,767	6.82	2	*rs80913047- rs81404614*	*rs80913047*	2.59E-07	*trans*	

Chromosomal location is based on *S*. *scrofa 11*.*1* assembly. Positions start and end refer to the eQTL interval. Gene annotation was performed considering one additional Mb at the start and at the end of the eQTL interval. Number of SNPs corresponds to the SNPs within the eQTL interval. P-value corresponds to the most significant SNP. For the *cis*-eQTL only the analyzed gene was considered.

*Genes with functions related to *IGF2*.

The SSC2 eQTL was divided in a *cis* and a *trans*-eQTL regions according to the distance from the *IGF2* gene. For the *cis*-eQTL region, where the *IGF2* gene was located, the *IGF2*:*g*.*3072G>A* mutation was the most associated SNP (p-value = 2.07x10^-15^). This result is in accordance with findings in muscle tissue, where I*GF2* mRNA expression was associated with this polymorphism [10] and suggests that it is also the causal mutation of *IGF2* gene expression in adipose tissue. A 25% of the phenotypic variance is explained by the *IGF2*:*g*.*3072G>A* polymorphism, indicating that other genetic variants and environmental factors are regulating *IGF2* gene expression in adipose tissue. In the same region, prior studies have reported the existence of *IGF2* antisense transcript in pigs and its coregulation with the *IGF2* gene in muscle and liver tissues. Furthermore, the antisense transcript was involved in the transcription regulation of *IGF2* promoters 2, 3 and 4 in post-natal muscle of animals carrying the *A* allele [29]. Therefore, it may be also involved in the regulation of *IGF2* in adipose tissue. In addition, *Splicing factor 1* (*SF1*) gene was mapped in the SSC2 *trans*-eQTL region (Table 1) and it was involved in the spliceosoma assembly and the alternative splicing which is an important mechanism for gene expression regulation [30].

Finally, *rs80913047* (p-value = 2.59x10^-7^) was the most significant associated SNP with BF *IGF2* expression on SSC8 but no candidate genes were annotated in this region.

eGWAS studies were also performed in animals of each backcross independently. In BC1_LD, no significant eQTLs regions were found (S1 Fig). This result is likely explained by the low number of animals with the *AA* genotype in the BC1_LD backcross (Table 2), being 0.2 the allele frequency of the *IGF2*:*g*.*3072A* allele.

**Table 2:** Summary of the number of animals used in this study.

		3BCs	BC1_LD	BC1_DU	BC1_PI
**Sex**	**Female**	186	65	63	58
	**Male**	169	49	59	61
**Genotype**	***AA***	131	4	61	66
	***GA***	148	39	56	53
	***GG***	76	71	5	0
**Paternal Allele**	***A***	145	23	61	61
	***G***	182	91	44	47

Number of animals are according to sex (n = 355), the *IGF2:g*.*3072G>A* polymorphism genotype (n = 355) and the paternal allele genotype (n = 355).

In addition, a previous work of our group performed in the *Longissimus dorsi* muscle of BC1_LD animals identified the *cis*-eQTL of the *IGF2* gene region, but the *IGF2*:*g*.*3072G>A* polymorphism was not the most significant SNP associated with the *IGF2* mRNA expression in muscle [20].

**Fig 2:**
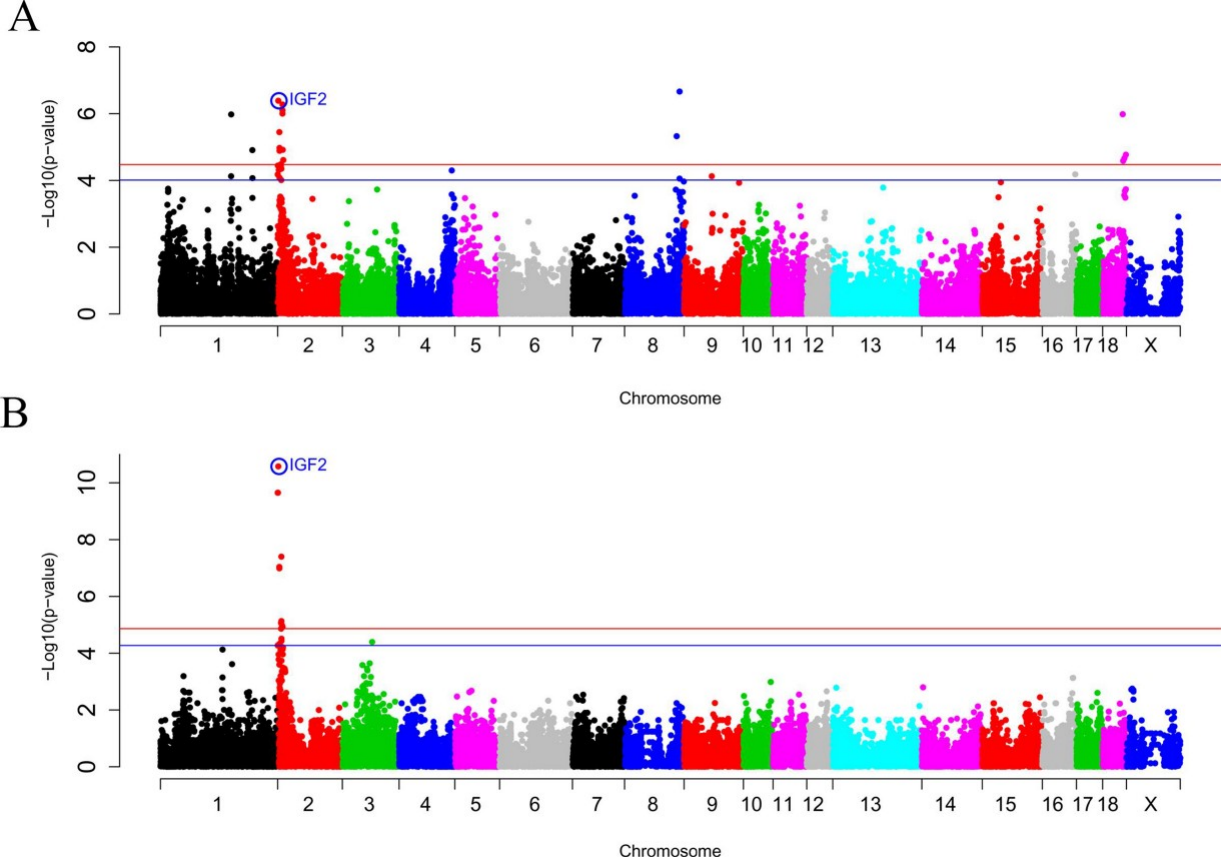
**GWAS plot of adipose tissue IGF2 gene expression in (A) BC1_DU and (B) BC1_PI.** Chromosome positions in Mb based on *S*. *scrofa 11*.*1* assembly of the pig genome are represented in the X-axis and the–log10 (p-value) is on the Y-axis. The red horizontal line indicates the genome-wide significant level (FDR-based *q*-value < 0.05) and the blue horizontal line represents the genome-wide suggestive level (FDR-based *q*-value <0.1). The *IGF2*:*g*.*3072G>A* polymorphism is circled and labelled as IGF2 in colour blue.

In contrast, the SSC2 and SSC8 eQTLs were also found in the BC1_DU backcross (Fig 2A), being the *rs81302016* SNP of the SSC8 the most significant associated SNP with the *IGF2* mRNA expression (p-value = 2.17x10^-7^). The *IGF2:g*.*3072G>A* polymorphism was the most associated SNP on SSC2 (p-value = 4.10x10^-7^). Moreover, a proximal region located at 11.6 Mb of SSC2 showed a strong signal, being *rs81336616* (p-value = 5.32x10^-7^) the second most significant SNP in this region. The *IGF2*:*g*.*3072G>A* polymorphism explains a 24% of the phenotypic variance of adipose tissue *IGF2* gene expression in BC1_DU. A linear mixed model using the *IGF2*:*g*.*3072G>A* polymorphism as a fixed effect was analysed, showing no other additional eQTL on SSC2 for *IGF2* gene expression (S2 Fig). However, a second eQTL at 11.6 Mb of SSC2 may not be discarded due to the linkage disequilibrium observed between *IGF2*:*g*.*3072G>A* and *rs81336616* SNPs in BC1_DU animals (R^2^ = 0.464, D' = 0.693). Moreover, two additional *trans*-eQTLs regions were detected at SSC1 and SSC18 in the BC1_DU population (Table 3).

**Table 3:** Significant eQTLs for adipose tissue *IGF2* gene expression in BC1_DU and BC1_PI.

Region	Chr	Start-End Positions	Size (Mb)	SNPs N	Start-End SNPs	Most Significant SNP	P-value	Type of eQTL	Candidate genes*
1_DU	1	165,536,222–215,454,308	49.92	4	*rs81349445- rs80815028*	*rs80815896*	1.05E-06	*trans*	
2_DU	2	0,145,257–13,156,928	13.01	23	*rs81306755- rs332366314*	*IGF2:g*.*3072G>A*	1.09E-06	*cis/trans*	*IGF2* and *SF1*
3_DU	8	121,609,989–138,238,585	16.63	4	*rs80913047- rs81406196*	*rs81302016*	2.17E-07	*trans*	
4_DU	18	46,815,469–54,261,734	7.45	4	*rs81470467- rs81471417*	*rs81470467*	1.04E-06	*trans*	*IGFBP1* and *IGFBP3*
1_PI	2	0,070,114–11,420,079	11.34	19	*rs81341288- rs81361529*	*IGF2:g*.*3072G>A*	2.64E-11	*cis/trans*	*IGF2* and *SF1*

Regions corresponding to BC1_DU and BC1_PI are referenced as _DU or _PI respectively. Chromosomal location is based on *S*. *scrofa 11*.*1* assembly. Positions start and end refer to the eQTL interval. Gene annotation was performed considering one additional Mb at the start and at the end of the eQTL interval. Number of SNPs corresponds to the SNPs within the eQTL interval. P-value corresponds to the most significant SNP. For the *cis*-eQTL only the analyzed gene was considered.

*Genes with functions related to *IGF2*.

Four significant associated SNPs were found in the SSC18 eQTL being *rs81470467* the most significant one (p-value = 1.04x10^-6^). Remarkably, two members of the IGF2 family were mapped in this region, the *insulin-like growth factor binding protein 1* and *3* (*IGFBP1* and *IGFBP3*), which are regulators of IGF activity, availability and tissue distribution [31]. Specifically, *IGFBP1* gene was involved in obesity prevention and developing glucose intolerance and *IGFBP3* was described as an inducer of insulin resistance [32].

Finally, in the BC1_PI backcross only the SSC2 eQTL was found (Fig 2B), being the *IGF2*g:3072G>A polymorphism the most significantly associated SNP with *IGF2* expression in adipose tissue (p-value = 2.64x10^-11^) (Table 3). The 46% of the phenotypic variance was explained by the *IGF2*:*g*.*3072G>A* polymorphism, a higher proportion than in the other backcrosses.

**Fig 3:**
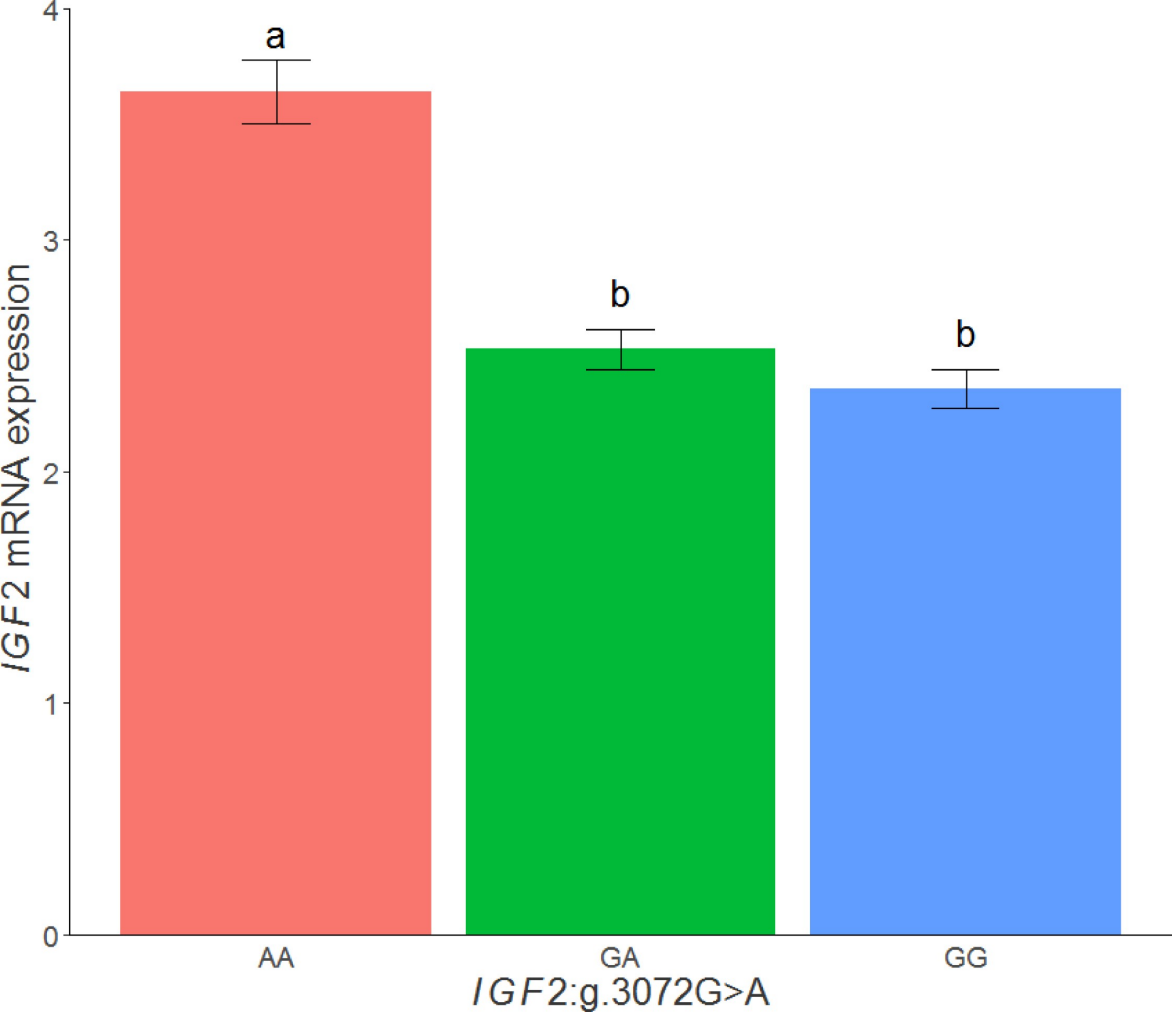
Plot of relative quantification of *IGF2* mRNA levels in adipose tissue of the 3BCs according to the *IGF2*:*g*.*3072G>A* SNP genotypes. Data represents means ± standard error of mean (SEM). Values with different superscript letters (a, b) indicate significant differences between groups (P-value <0.05).

When the *IGF2* expression values in adipose tissue of the 3BCs were classified according to the *IGF2*:*g*.*3072G>A* genotypes, animals with the *AA* genotype (mean = 3.64, n = 131) showed the highest mRNA expression with significant differences with *GA* (mean = 2.53, n = 148) and *GG* (mean = 2.36, n = 75) genotypes (*AA*-*GA*: p-value = 1.78x10^-13^, *AA*-*GG*: p-value = 7.25x10^-04^, *GA*-*GG*: p-value = 9.9x10^-02^) (Fig 3). Similar results were observed when the three backcrosses were analyzed separately.

### Analysis of the imprinting effect on *IGF2* gene expression

**Fig 4:**
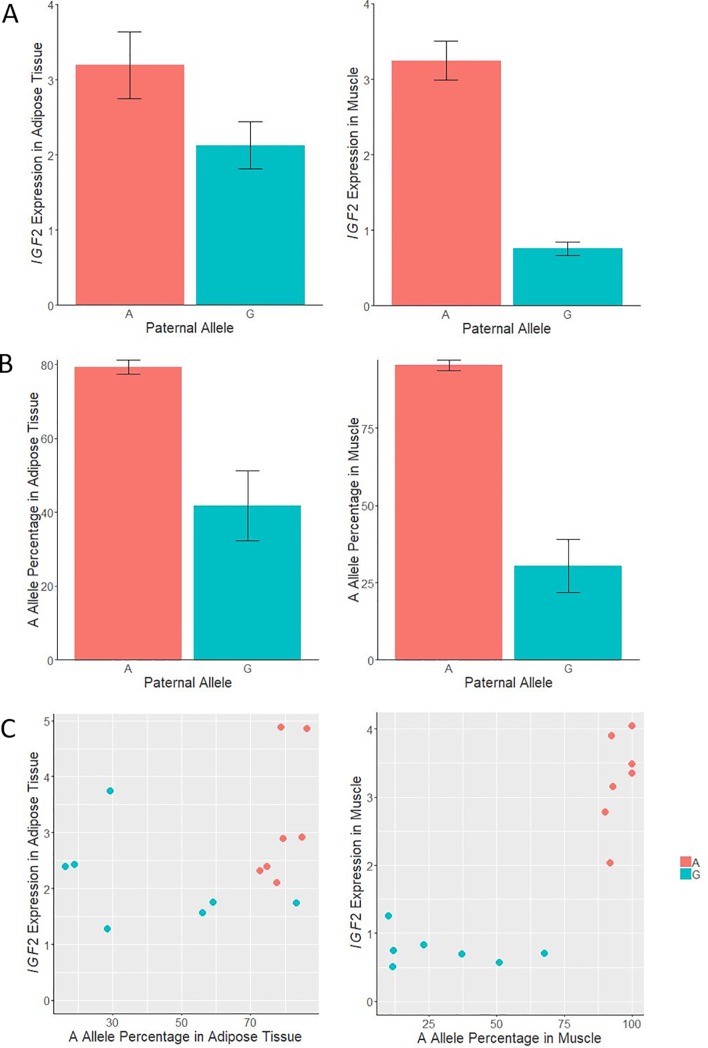
Plots of relative quantification of *IGF2* gene expression and allele percentage in muscle and adipose tissue according to the inherited paternal allele, and scatterplot combining *IGF2* gene expression and allele percentage in both tissues according to the paternal allele. The analysis was done in animals where paternally inherited allele was deduced. Data of *IGF2* gene expression represents means ± standard error of mean (SEM). Data for *A* allele are presented as percentage ± standard error.

To investigate the *IGF2* gene imprinting in adipose tissue, pyrosequencing analysis was performed in animals with known paternally-inherited alleles, both in muscle and adipose tissues (Fig 4). *IGF2* gene expression in both tissues was also obtained for these animals. As expected, in muscle the *A* allele percentage (from the sum of the two alleles) was higher (95.4%) in animals inheriting the *A* allele from his father than in animals inheriting the *G* allele (30.4%; p-value = 7.27x10^-06^). In adipose tissue, the *A* allele percentage was also higher (79.3%) in animals inheriting the *A* allele from his father than in animals receiving the *G* allele (41.7%; p-value = 0.002). According to these results, there is imprinting of the *IGF2* gene in both tissues, although stronger differences among the paternally and maternally inherited alleles were observed in muscle.

**Fig 5:**
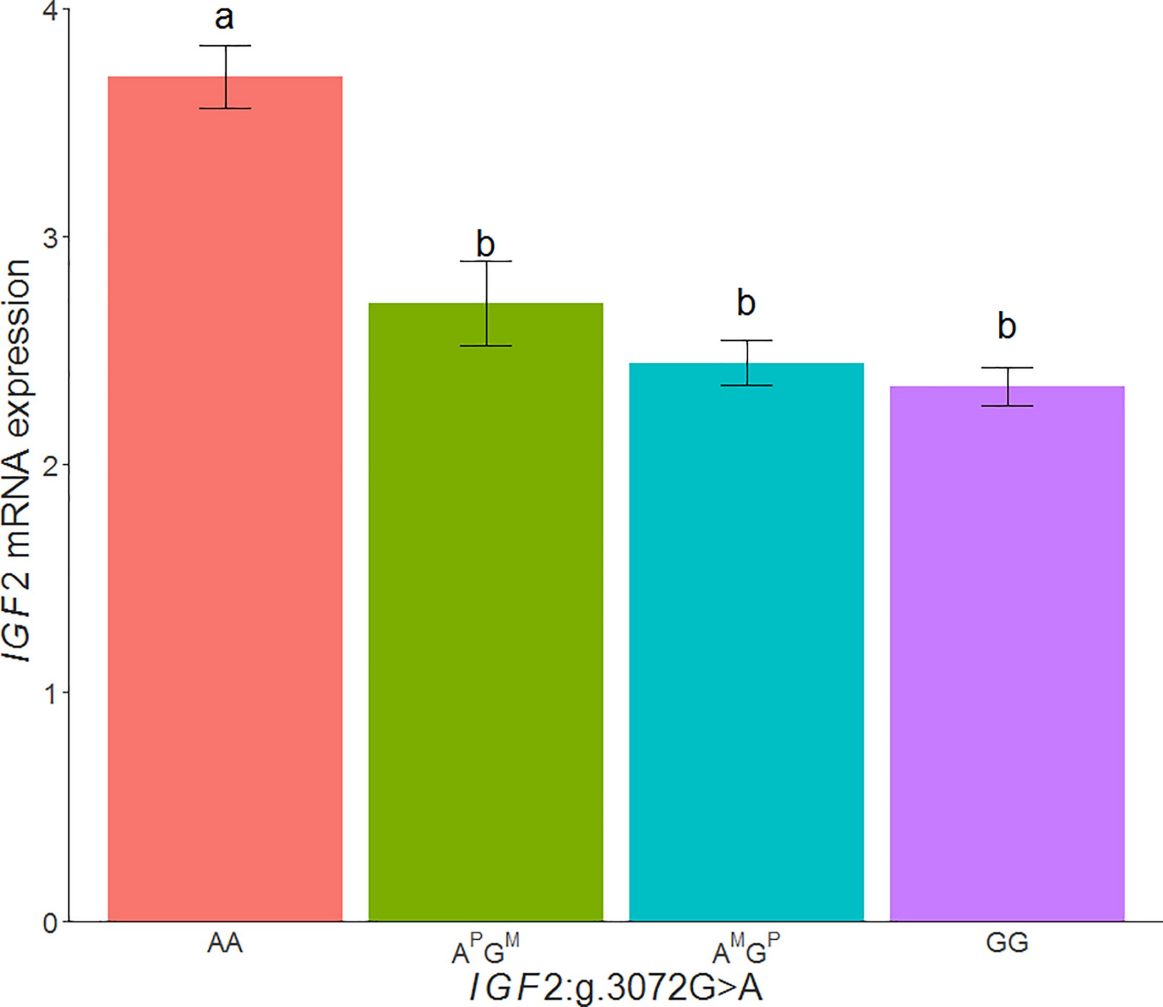
Plot of relative quantification of *IGF2* mRNA levels in adipose tissue according to the genotype of the *IGF2*:*g*.*3072G>A* polymorphism. The analysis was done in animals where paternally inherited allele was deduced, *A*^*P*^*G*^*M*^ means a paternally inherited *A* allele and maternal inherited *G* allele, on the contrary, *A*^*M*^*G*^*P*^ represents a maternal inherited *A* allele and paternal inherited *G* allele. Data represents means ± standard error of mean (SEM).

However, these results may not agree with the adipose tissue *IGF2* gene expression comparison between the *A*^*P*^*G*^*M*^ and *A*^*M*^*G*^*P*^ genotypes (p-value = 1.90x10^-01^), in which no significant differences between these genotypes was observed, when the paternally inherited allele was deduced from the genotypes of the parents in 355 backcrossed animals. Conversely, the animals with the *AA* genotype showed a higher *IGF2* gene expression in comparison with the other genotypes (Fig 5). Nonetheless, these results can be explained by a higher expression of the G allele in adipose tissue, in comparison with muscle, which may be in turn produced by a reduction of binding of the ZBED6 repressor.

The structure of the pig *IGF2* gene consists in 10 exons but the mature form only contains the last three. The other exons, along with the four promoters included in the gene, are involved in the *IGF2* expression in a tissue specific manner [33]. For example, *IGF2* promoter 1 is used in liver instead of promoters 2, 3 and 4 that are used in muscle, being promoters tissue-dependent. Epigenetic regulation mechanisms, like imprinting status and its reflection in DNA methylation patterns are also completely different in each tissue [10], although no studies have been done in adipose tissue either in humans and pigs [34]. It has been reported that *IGF2* is expressed from both parental alleles in liver, whereas imprinting has been described in mesodermal tissues such as skeletal muscle and kidney in foetal and adult animals [35]. Thus, we could assume that all the tissues coming from the mesoderm, including the adipose tissue, should present the same imprinting pattern. Supporting this hypothesis our results showed imprinting of the *IGF2* gene in muscle and adipose tissues. However, further studies are required to deepen the mechanism of regulation of *IGF2* gene expression in adipose tissue, which seems to play an important role in this tissue.

### Sex and breed effects on *IGF2* gene expression

In order to identify if *IGF2* expression presents sexual dimorphism, the *IGF2* mRNA levels measured in adipose tissue of the 3BCs animals were analyzed according to sex. The obtained results showed that gene expression was higher in males (mean = 3.12, SD = 1.41, n = 169) than in females (mean = 2.71, SD = 1.26, n = 185), with significant differences (p-value = 1.19x10^-4^) and genotypic frequencies were balanced in the two sexes (Table 2). It is reported that some imprinted genes are related with sexual dimorphism in mice, including *IGF2*, in which gene expression is also higher in males than females and this can led to differences in body size between sexes [36].

Concerning the backcross effect, the highest *IGF2* gene expression was observed in BC1_DU (mean = 3.66, SD = 1.68) followed by BC1_PI (mean = 2.64, SD = 1.08) and BC1_LD (mean = 2.36, SD = 0.71). Significant differences were found between BC1_DU and BC1_LD (p-value = 1.66x10^-3^), and when comparing BC1_DU and BC1_PI (p-value = 1.55x10^-5^). On the contrary, no significant differences were obtained when gene expression of BC1_PI and BC1_LD was compared. These results are in accordance with the study of Redjuch *et al*. (2010), in which animals from Duroc, Large White, and Landrace breeds carrying the paternally derived *A* allele presented differences in *IGF2* gene expression, being higher in Duroc [37]. Hence, the differential *IGF2* gene expression among backcrosses may be explained by differences in genotypic frequencies (Table 2). Animals carrying the paternally derived *A* allele, that was deduced from the genotypes of the parents, were also analyzed according to the breed effect. The same results were obtained: highest *IGF2* gene expression corresponded to BC1_DU (mean = 4.30, SD = 1.77), followed by BC1_PI (mean = 2.95, SD = 1.05) and BC1_LD (mean = 2.55, SD = 0.68) and significant differences were observed between the same breeds than in the previous study.

### Association study for adipose tissue fatty acid composition and SSC2 polymorphisms

**Fig 6:**
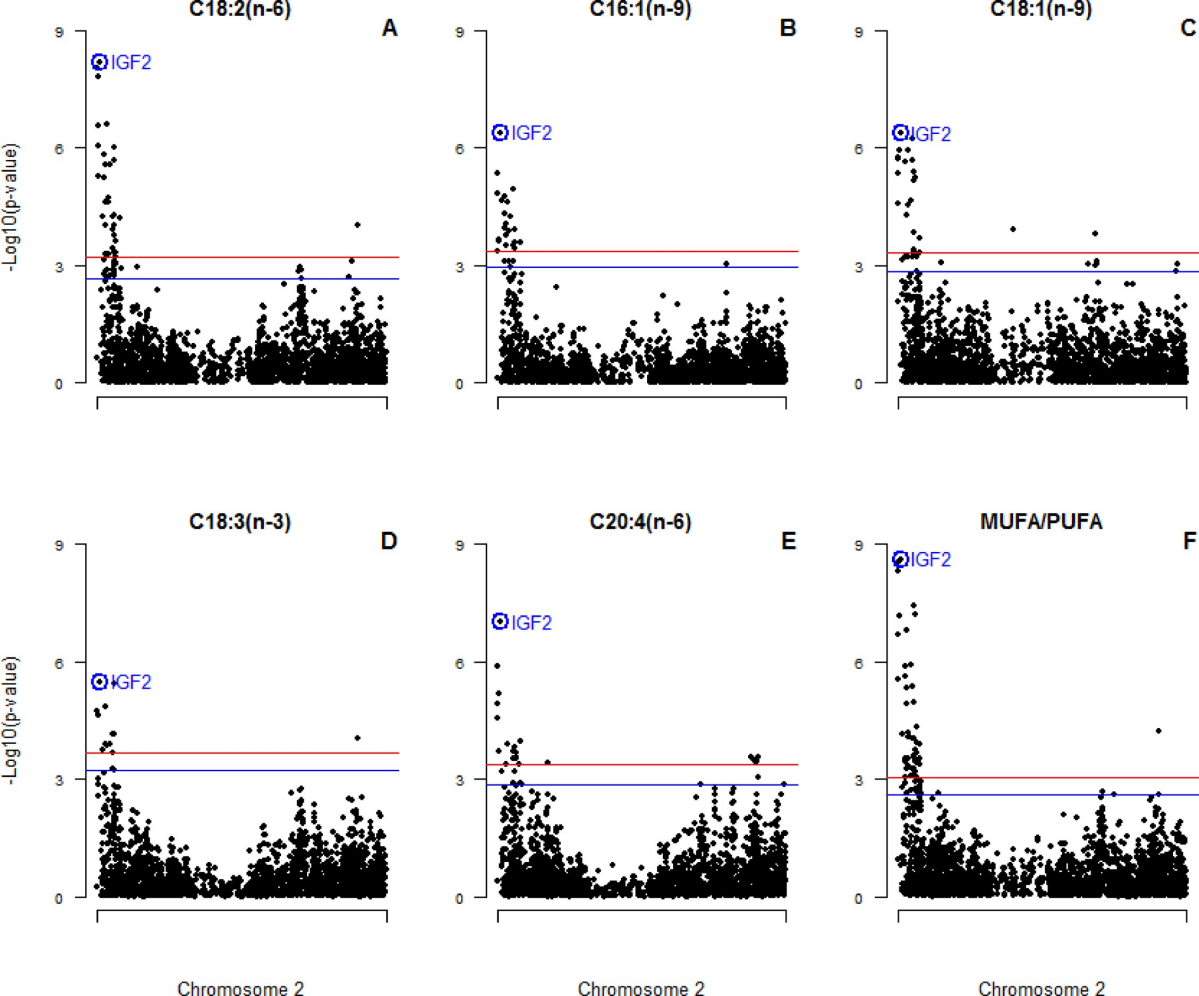
Plot of SSC2 SNPs association for significant FAs. (A) linoleic acid, (B) hexadecanoic acid, (C) oleic acid, (D) α-linoleic acid, and (E) arachidonic acid, and (F) MUFA/PUFA ratio in adipose tissue in 3BCs. Chromosome 2 (SSC2) positions in Mb based on *S*. *scrofa 11*.*1* assembly of the pig genome are represented in the X-axis and the–log10 (p-value) is on the Y-axis. The red horizontal line indicates the chromosomal-wide significant level (FDR-based *q*-value < 0.05) and the blue horizontal line represents the genome-wide suggestive level (FDR-based *q*-value < 0.1). The *IGF2*:*g*.*3072G>A* polymorphism is circled and labelled as IGF2 in colour blue.

Since the identification of *IGF2*:*g*.*3072G>A* substitution as the causal mutation of the imprinted QTL for muscle growth, fat deposition and heart size [10] several association studies between the polymorphism and growth traits have been performed in different populations [14–16]. However, the association with FA composition in adipose tissue of the *IGF2*:*g*.*3072G>A* polymorphism has not been tested. In the present work, association analyses were carried out among 2,431 SNPs of SSC2, including the *IGF2*:*g*.*3072G>A* polymorphism, and FA composition measured in adipose tissue. The *IGF2*:*g*.*3072G>A* polymorphism was the most significantly associated with linoleic (C18:2(n-6); p-value = 6.44x10^-09^), hexadecanoic (C16:1(n-9); p-value = 4.04x10^-07^), oleic (C18:1(n-9); p-value = 4.18x10^-07^), α-linoleic (C18:3(n-3); p-value = 3.30x10^-06^), arachidonic (C20:4(n-6); p-value = 9.82x10^-08^) FAs and the MUFA/PUFA ratio (p-value = 2.51x10^-9^) (Fig 6).

Correlations were performed between *IGF2* expression and FA composition measured in BF to deepen the relationship between gene expression and phenotypes. Our results showed a low correlation between the *IGF2* gene expression and FA composition. In general, a positive correlation between *IGF2* gene expression and the proportion of essential FAs, such as linoleic (r = 0.21, p-value = 4.92x10^-04^) and α-linoleic (r = 0.19, p-value = 3.56x10^-04^) FAs, in adipose tissue was observed. Conversely, a negative correlation with oleic FA (r = -0.21, p-value = 5.78x10^-05^) was shown. The imprinting model was also tested for FA composition and we neither could see FA content significant difference in the heterozygous genotype depending on which allele comes from the father.

SSC2 association studies were also performed independently in each backcross. In BC1_LD the *IGF2*:*g*.*3072G>A* polymorphism was not significantly associated with FA composition, and this could be also explained by the differences in the allele frequency of the SNP explained before. However, other significant polymorphisms of SSC2 were identified for linoleic (*rs81355859*, p-value = 3.22x10^-07^), hexadecanoic (*rs81322199*, p-value = 9.63x10^-07^), oleic (*rs81287787*, p-value = 4.39x10^-06^) and α-linoleic (*rs81316644*, p-value = 8.04x10^-06^) acids as well as for the MUFA/PUFA ratio (*rs81355859*, p-value = 1.02x10^-06^) (S3 Fig). In BC1_DU, there were not significant polymorphisms associated with the FA composition measured in adipose tissue (S4 Fig). Finally, in BC1_PI the *IGF2*:*g*.*3072G>A* polymorphism was the most significant associated SNP with linoleic (C18:2(n-6); p-value = 1.79x10^-05^), oleic (C18:1(n-9); p-value = 1.04x10^-07^), and arachidonic (C20:4(n-6); p-value = 2.79x10^-05^) FAs and the MUFA/PUFA ratio (p-value = 1.67x10^-7^) (S5 Fig), while the SNP *rs81312355* was the most significant associated polymorphism for the α-linoleic FA (p-value = 3.80x10^-05^).

The comparison among the 3BCs and the backcross specific studies showed that the *IGF2*:*g*.*3072G>A* polymorphism was not always the most significant SSC2 SNP. In BC1_LD animals other SNPs were more significant, in BC1_DU animals no significant SNPs were identified, and in BC1_PI animals *IGF2*:*g*.*3072G>A* was only the most significant SNP in four of the six traits described in the 3BCs animals. These results suggest that other variants associated with adipose tissue FA composition are segregating in specific backcrosses, mainly in BC1_LD.

Little is known about the relationship between the *IGF2* gene and lipid metabolism but it has been described that the IGF2 mRNA binding protein p62/IGF2BP2-2 is related with FA elongation in human liver disease [38]. Adipose tissue, which is the principal organ involved in the FA synthesis [39], has a high FA content, specifically of PUFA such as linoleic and α-linoleic FAs. These essential FAs are only provided by the diet and are readily stored in adipose tissue [40]. Besides, it was reported that there is an inverse relationship between the amount of α-linoleic in BF and the BF thickness, and this trait is related to fat quality in terms of firmness and the degree of cohesiveness within lean and fat tissues [2]. On the other hand, oleic acid is the most abundant MUFA in pork, comprising nearly 35%-45% of total FAs content. It is associated with consumer’s acceptability of high quality cured products, in terms of organoleptic, technological and nutritional values of meat [41]. To study the effect of *IGF2*:*g*.*3072G>A* polymorphism in BF thickness, an association study was performed in 330 3BCs animals. No significant associations were found between the *IGF2*:*g*.*3072G>A* polymorphism and the fat measure (S6 Fig). The most significant SNP on SSC2 was *rs81214179*, which is located at 8.9 Mb (p-value = 1.57x10^-06^), where three desaturases involved in the synthesis of highly unsaturated FAs from essential FAs provided by the diet [40] were mapped: *fatty acid desaturase 1* (*FADS1*), *fatty acid desaturase 2* (*FADS2*) and *fatty acid desaturase 3* (*FADS3*).

In summary, according to the *IGF2*:*g*.*3072G>A* polymorphism, homozygous *AA* animals presented the highest *IGF2* gene expression in adipose tissue, a higher percentage of PUFA and a lower MUFA content in comparison to the other two genotypes. The association of the *IGF2*:*g*.*3072G>A* polymorphism with some relevant FAs suggest that IGF2 plays a role in the variability of FA composition in adipose tissue. Nevertheless, we cannot exclude that other proximal genetic variants, such as polymorphisms located in the desaturases genes, are affecting FA content. Hence, further works are required to deepen the study of this complex SSC2 region. Considering that some studies in human reported the involvement of IGF2 in the metabolism and body fat composition, this gene could also play a physiological role in pig adipose tissue.

## Conclusions

In the present work, the *AA* genotype *IGF2*:*g*.*3072G>A* polymorphism has been associated with a higher *IGF2* gene expression in BF adipose tissue. In addition, the *IGF2* gene expression in adipose tissue is explained by an imprinting model. Finally, the polymorphism was significantly associated with FA composition measured in BF and animals carrying the *A* allele showed a higher PUFA and lower MUFA content, although there may be other genetic variants affecting FA content. Hence, *IGF2* gene can play a relevant role in pig adipose tissue.

## Supporting information

S1 Table Primers used for IGF2 gene expression quantification by RT-qPCR. (XLSX)

S1 Fig GWAS plot of adipose tissue *IGF2* gene expression in BC1_LD. Chromosome positions in Mb based on *S*. *scrofa 11*.*1* assembly of the pig genome are represented in the X-axis and the–log10 (p-value) is on the Y-axis. The *IGF2*:*g*.*3072G>A* polymorphism is circled and labelled as IGF2 in colour blue.
(TIF)

S2 Fig GWAS plot of adipose tissue *IGF2* gene expression in BC1_DU using *IGF2*:*g*.*3072G>A* polymorphism as a fixed effect. Chromosome positions in Mb based on *S*. *scrofa 11*.*1* assembly of the pig genome are represented in the X-axis and the–log10 (p-value) is on the Y-axis. The red horizontal line indicates the genome-wide significant level (FDR-based *q*-value < 0.05) and the blue horizontal line represents the genome-wide suggestive level (FDR-based *q*-value <0.1).
(TIF)

S3 Fig Plot of SSC2 SNPs association for significant FAs in BC1_LD. (A) linoleic acid, (B) hexadecanoic acid, (C) oleic acid, (D) α-linoleic acid, and (E) arachidonic acid, and (F) MUFA/PUFA ratio in adipose tissue in 3BCs. Chromosome 2 (SSC2) positions in Mb based on *S*. *scrofa 11*.*1* assembly of the pig genome are represented in the X-axis and the–log10 (p-value) is on the Y-axis. The red horizontal line indicates the chromosomal-wide significant level (FDR-based *q*-value < 0.05) and the blue horizontal line represents the genome-wide suggestive level (FDR-based *q*-value < 0.1). The IGF2:*g*.*3072G>A* polymorphism is circled and labelled as IGF2 in colour blue.
(TIF)

S4 Fig Plot of SSC2 SNPs association for significant FAs in the BC1_DU. (A) linoleic acid, (B) hexadecanoic acid, (C) oleic acid, (D) α-linoleic acid, and (E) arachidonic acid, and (F) MUFA/PUFA ratio in adipose tissue in 3BCs. Chromosome 2 (SSC2) positions in Mb based on *S*. *scrofa 11*.*1* assembly of the pig genome are represented in the X-axis and the–log10 (p-value) is on the Y-axis. The red horizontal line indicates the chromosomal-wide significant level (FDR-based *q*-value < 0.05) and the blue horizontal line represents the genome-wide suggestive level (FDR-based *q*-value < 0.1). The *IGF2*:*g*.*3072G>A* polymorphism is circled and labelled as IGF2 in colour blue.
(TIF)

S5 Fig Plot of SSC2 SNPs association for significant FAs in the BC1_PI. (A) linoleic acid, (B) hexadecanoic acid,(C) oleic acid, (D) α-linoleic acid, and (E) arachidonic acid, and (F) MUFA/PUFA ratio in adipose tissue in 3BCs. Chromosome 2 (SSC2) positions in Mb based on *S*. *scrofa 11*.*1* assembly of the pig genome are represented in the X-axis and the–log10 (p-value) is on the Y-axis. The red horizontal line indicates the chromosomal-wide significant level (FDR-based *q*-value < 0.05) and the blue horizontal line represents the genome-wide suggestive level (FDR-based *q*-value < 0.1). The *IGF2*:*g*.*3072G>A* polymorphism is circled and labelled as IGF2 in colour blue.
(TIF)

S6 Fig GWAS plot of BF thickness measure in the 3BCs animals. Chromosome positions in Mb based on *S*. *scrofa 11*.*1* assembly of the pig genome are represented in the X-axis and the–log10 (p-value) is on the Y-axis. The red horizontal line indicates the genome-wide significant level (FDR-based *q*-value < 0.05) and the blue horizontal line represents the genome-wide suggestive level (FDR-based *q*-value <0.1).
(TIF)
