# Supplementary figures and images for: Analysis of porcine IGF2 gene expression in adipose tissue and its effect on fatty acid composition
Source: PLoS One. 2019 Aug 8;14(8):e0220708. doi: 10.1371/journal.pone.0220708 (PMC6687288; doi:10.1371/journal.pone.0220708)

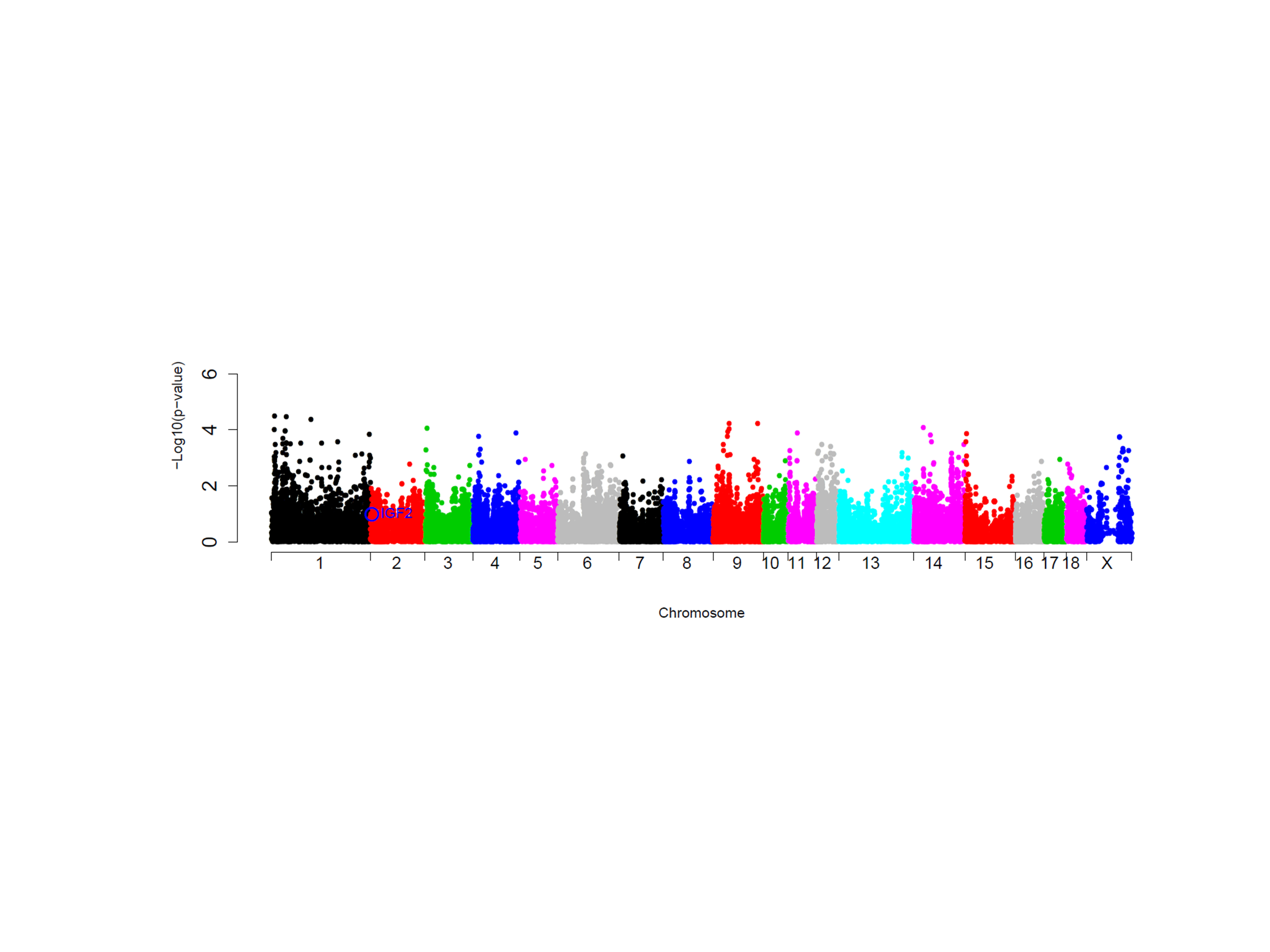

Supplement: S1 Fig — Chromosome positions in Mb based on S. scrofa 11.1 assembly of the pig genome are represented in the X-axis and the–log10 (p-value) is on the Y-axis. The IGF2:g.3072G>A polymorphism is circled and labelled as IGF2 in colour blue. (TIF) [file pone.0220708.s002.tif]

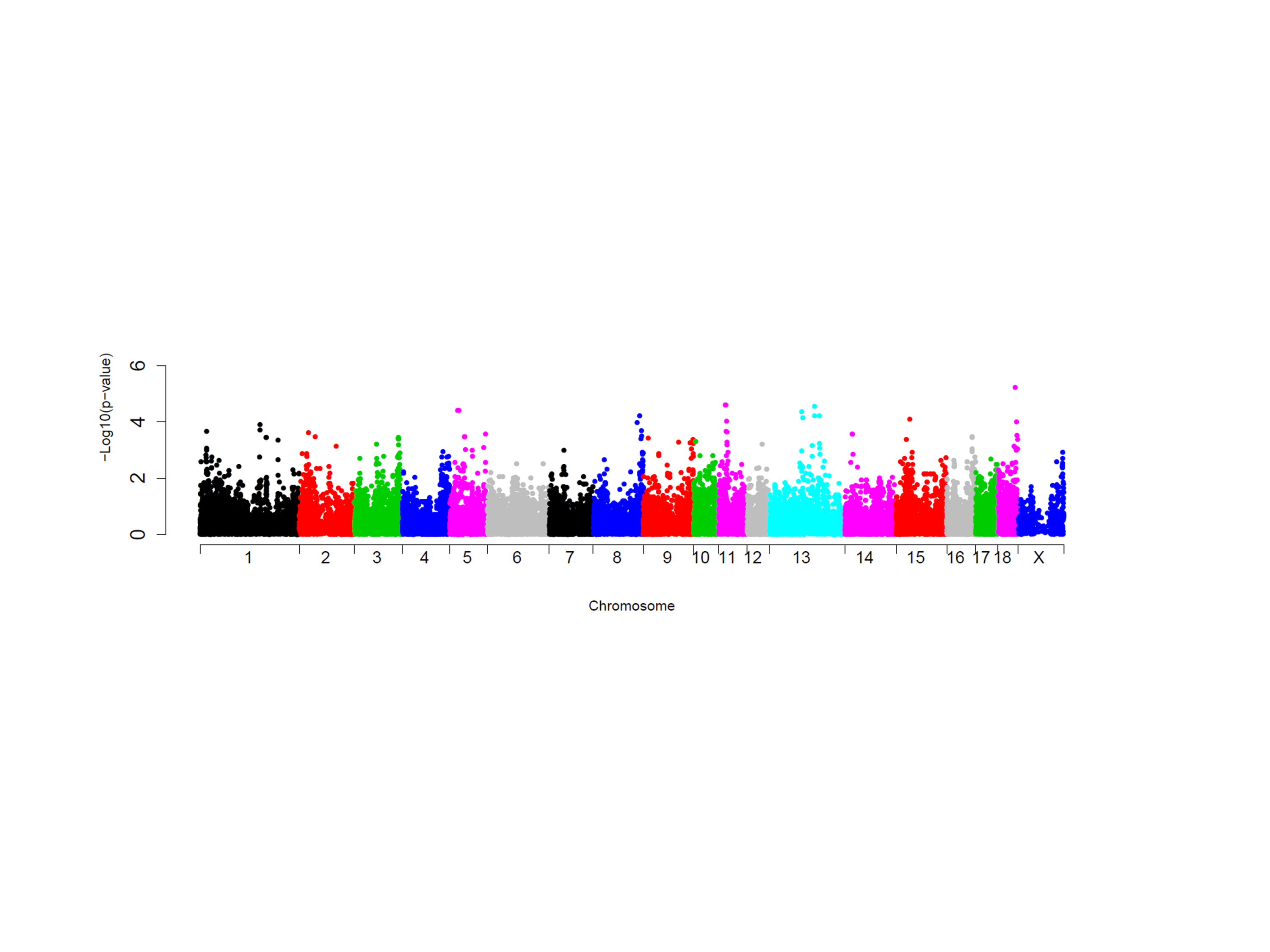

Supplement: S2 Fig — Chromosome positions in Mb based on S. scrofa 11.1 assembly of the pig genome are represented in the X-axis and the–log10 (p-value) is on the Y-axis. The red horizontal line indicates the genome-wide significant level (FDR-based q-value < 0.05) and the blue horizontal line represents the genome-wide suggestive level (FDR-based q-value <0.1). (TIF) [file pone.0220708.s003.tif]

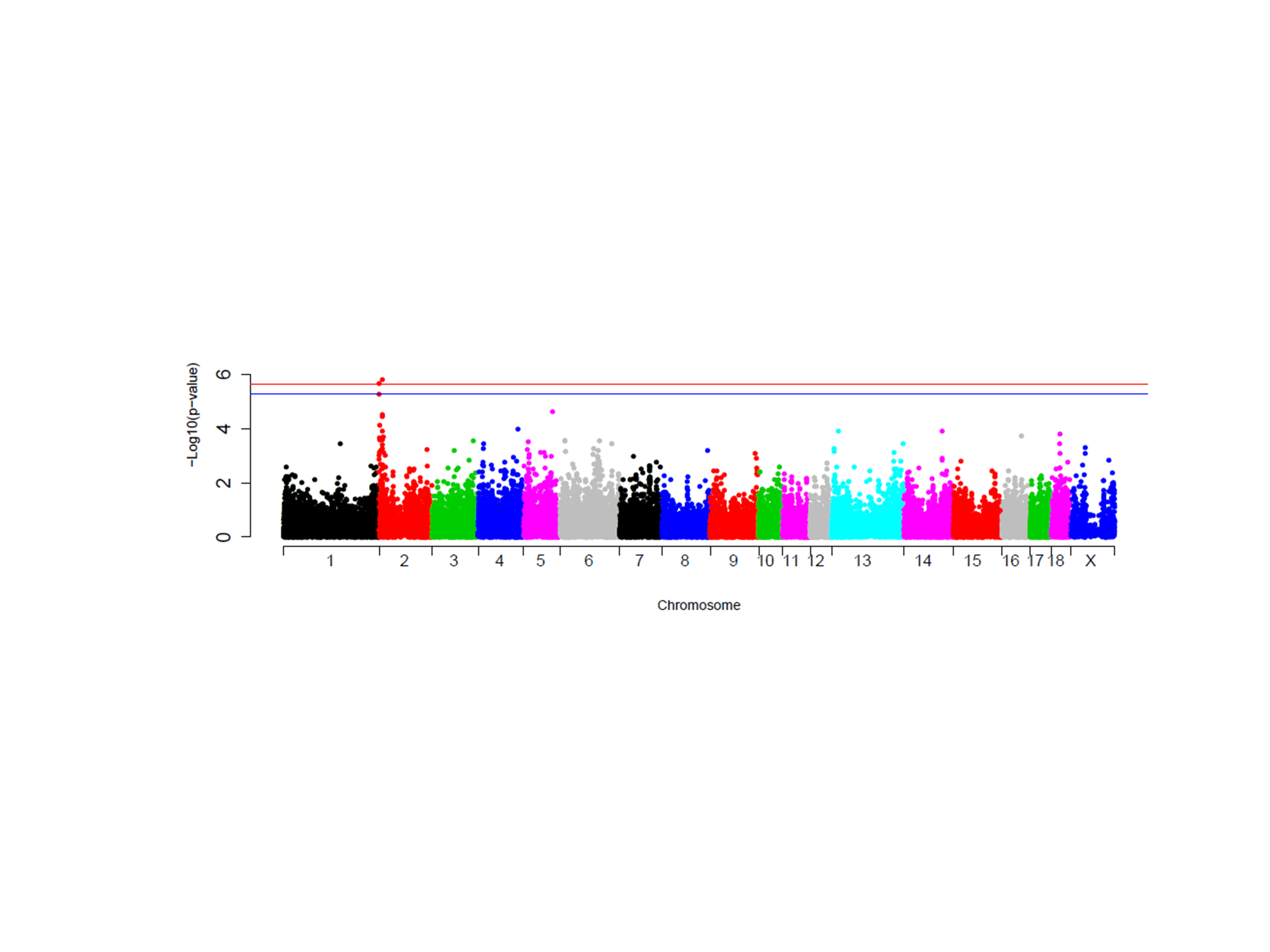

Supplement: S6 Fig — Chromosome positions in Mb based on S. scrofa 11.1 assembly of the pig genome are represented in the X-axis and the–log10 (p-value) is on the Y-axis. The red horizontal line indicates the genome-wide significant level (FDR-based q-value < 0.05) and the blue horizontal line represents the genome-wide suggestive level (FDR-based q-value <0.1). (TIF) [file pone.0220708.s007.tif]
